# The *Helicobacter pylori* type IV secretion system promotes IL-8 synthesis in a model of pediatric airway epithelium via p38 MAP kinase

**DOI:** 10.1371/journal.pone.0183324

**Published:** 2017-08-15

**Authors:** Myra G. dela Pena-Ponce, Monica T. Jimenez, Lori M. Hansen, Jay V. Solnick, Lisa A. Miller

**Affiliations:** 1 California National Primate Research Center, University of California Davis, Davis, California, United States of America; 2 Center for Comparative Medicine, University of California Davis, Davis, California, United States of America; 3 Departments of Medicine and Microbiology & Immunology, School of Medicine, University of California Davis, Davis, California, United States of America; 4 Department of Anatomy, Physiology and Cell Biology, School of Veterinary Medicine, University of California Davis, Davis, California, United States of America; La Trobe University College of Science Health and Engineering, AUSTRALIA

## Abstract

Epidemiologic studies have reported an inverse relationship between childhood *Helicobacter pylori* infection and development of allergic asthma. Because lung epithelium plays an important role in allergic asthma pathogenesis, we hypothesized that *H*. *pylori* may directly influence airway epithelial cell innate immune function, particularly in early childhood. To test our hypothesis, we established an *in vitro H*. *pylori* infection model using primary tracheobronchial epithelial cell cultures derived from infant, juvenile and adult rhesus monkeys. Airway epithelial cell cultures were infected with wild-type or cag pathogenicity island mutant *H*. *pylori* strains, followed by evaluation of IL-8 and IL-6 protein synthesis. We found that *H*. *pylori* primarily increased IL-8 synthesis in a MOI and age-dependent fashion, with a greater than 4-fold induction in infant versus adult cultures. *H*. *pylori*-induced IL-8 synthesis in infant and juvenile cultures was significantly reduced by cag pathogenicity island mutants, indicating a requirement for the type IV secretion system. Although peptidoglycan recognition of nucleotide binding oligomerization domain-containing protein 1 (NOD1) and NF-kappaB have been implicated as key cytokine signaling molecules for *H*. *pylori* infection in gastric epithelium, NOD1 (ML130) or NF-kappaB (JSH-23) inhibitors minimally affected IL-8 synthesis in airway epithelial cell cultures following *H*. *pylori* infection. In contrast, inhibition of the p38 MAP kinase pathway (SB203580) resulted in almost complete suppression of *H*. *pylori*-induced IL-8 synthesis. Collectively, these results indicate that *H*. *pylori* can preferentially elicit IL-8 synthesis in a model of pediatric airway epithelium using the type IV secretion system via p38 MAP kinase.

## Introduction

*Helicobacter pylori* (*H*. *pylori*) is the most common cause of bacterial infection in the human gastrointestinal tract, leading in some patients to peptic ulcer disease or gastric adenocarcinoma. Human colonization with this gram-negative bacterium has been dated to approximately 60,000 years ago; the successful survival of *H*. *pylori* is likely due to its ability to both evade and subvert the host immune system [1]. The frequency of *H*. *pylori* infected humans from industrialized countries has progressively declined as rates of chronic inflammatory diseases increase, leading to the speculation that *H*. *pylori* may have a protective role in human health. This hypothesis has been supported by multiple cross-sectional and independent epidemiologic studies that have suggested early life *H*. *pylori* infection can prevent the development of childhood asthma, allergy and other atopic diseases [2–4]. How a gut microbe such as *H*. *pylori* might confer protection against asthma development in young individuals is not well understood. Experiments in mice suggest that regulatory T cells induced by *H*. *pylori* are highly suppressive in neonates and appear to block pathogenic effector T cells from causing damage in the respiratory tract [5]. There is also evidence that the *H*. *pylori* cytotoxin VacA can be aspirated into the airways and cause pleiotropic effects on lung epithelial cells via induction of proinflammatory cytokines [6]. Whether *H*. *pylori* may benefit respiratory health by immune activation of lymphocyte populations or direct influence on lung epithelium is yet to be determined.

The epithelial lining of the lung is traditionally viewed as a physical barrier that protects the host from inhaled substances coming from the external environment and eliminates materials through mucociliary clearance mechanisms. However, there is growing evidence that mediators produced by airway epithelium have antimicrobial and immunoregulatory properties that are essential for host pathogen defense. The airway epithelium itself expresses pathogen-recognition receptors (PRRs) to detect an array of pathogen-associated molecular patterns (PAMPS) from microbes [7]. For example, in *Staphylococcus aureus* infection the airway epithelium recognizes bacterial components via activation of Toll-like receptors (TLR), which subsequently activate signaling cascades that promote expression of Type I interferons and proinflammatory cytokines [8, 9]. After infection with *Mycobacterium tuberculosis*, expression of IL-8 by airway epithelial cells is enhanced following activation of IL-1 cytokine networks by monocytes, suggesting the capacity for regulatory cross-talk between the epithelium and hematopoietic cells within the lung [10]. A link between environmental microbe exposure and childhood asthma has been reported in human cohort studies [11–13], and experimental animal models indicate that expression of the lipopolysaccharide receptor TLR4 on airway epithelium is essential for establishment of the allergic asthma phenotype, suggesting that the presence of certain gram-negative bacteria in the lung may also contribute to chronic disease [13].

It is clear that airway epithelium has a multifaceted barrier role in the respiratory tract, where it both initiates and augments mechanisms of host pathogen defense [14]. However, our understanding of immunity within the context of the infant lung mucosa is limited, largely due to ethical challenges of obtaining samples from human neonates. It is well known that susceptibility to infectious disease during infancy is due to a functionally immature immune system relative to that of adults [15–19]. Using the rhesus macaque monkey as a model for pediatric lung development, we have shown that the innate immune responses of airway epithelial cells from infant monkeys are functionally attenuated relative to adult counterparts when exposed to either endotoxin or H1N1 infection [20, 21]. Our findings to date suggest that airway epithelial cells in the infant monkey contribute to the known predisposition of pediatric subjects to infection by respiratory pathogens, but it is unknown whether this susceptibility also influences long term health outcomes.

Because asthma protection conferred by *H*. *pylori* has been linked to childhood asthma and allergy, we hypothesized that *H*. *pylori* can not only directly elicit airway epithelial cell innate immune responses, but does so in an age-dependent fashion. We utilized the rhesus macaque as it provides an excellent model system for studying immunomodulation of airway epithelium as a function of age. Our results show that infant and juvenile airway epithelium is distinctly susceptible to the proinflammatory effects of *H*. *pylori*, and that the cellular mechanism appears to utilize the p38-MAPK pathway. Determining how *H*. *pylori* directly modulates the airways during childhood development may have implications for how *H*. *pylori* infection can have a protective role in preventing allergy and asthma later in life.

## Methods

### Ethics statement

Tissue specimens from nonhuman primates were obtained post-mortem from Pathology, a unit of Primate Services at the California National Primate Research Center (CNPRC). Animals were not a part of an experimental research protocol. The CNPRC vivarium is a component of the UC Davis Association for Assessment and Accreditation of Laboratory Animal Care International (AAALAC)-accredited program. At UC Davis, a single Institutional Animal Care and Use Committee (IACUC) oversees all animal use in research and teaching to ensure that the highest ethical and animal welfare standards are met. The CNPRC vivarium is approved by the UC Davis IACUC.

### Airway epithelial cell culture

The papilloma virus-immortalized human bronchial epithelial cell line 16-HBE, was used for initial verification of *H*. *pylori* immune induction in airway [22]. 16-HBE cells were plated in 24 well polystyrene cell culture cluster plates (Corning, Corning, NY) at a density of 4x10^5^ cells per well and cultured in serum-free airway epithelial cell media supplemented with growth factors (BEGM, Lonza, Walkersville, MD) and retinoic acid (50nM Sigma-Aldrich Corp., St. Louis, MO).

Primary rhesus macaque monkey airway epithelial cells were cultured as previously described [21]. In brief, airway epithelial cells were isolated from trachea collected from infant (6–10 months), juvenile (1–2 yr.) or adult monkeys (6–8 yr.) using protease digestion methods [20]. Isolated epithelial cells were plated at a density of 1x10^5^ cells per 6.5mm, 0.4 um pore size transwell clear polyester membrane insert (Corning, Corning, NY) coated with FNC coating mix (Athena Enzyme Systems, Baltimore, MD). Cells were first expanded in BEGM supplemented with retinoic acid until confluent (approximately 2 weeks), then cultured under air-liquid interface (ALI) conditions for an additional 7 days to achieve differentiation using previously established methods [23, 24]. Polarization of cultures was confirmed by measurement of trans-epithelial resistance using an STX2 Electrode (World Precision Instruments Inc., Sarasota, FL).

### Bacterial strains and culture

*H*. *pylori* strains J166 and PMSS1 are wild type human isolates that express the virulence factor *cagA* encoded within the *cag* pathogenicity island (*cag*PAI). Knockouts for *cagA* and *cagY* in these strains were constructed by an allelic exchange method as previously described [25]. All strains used for this study are listed in Table 1, along with their characteristics. Bacteria cultures were performed on brucella agar (BBL/Becton Dickinson, Sparks, MD) supplemented with 5% heat-inactivated newborn calf serum (Invitrogen, Carlsbad, CA) and TVPA antibiotics (trimethoprim, 5 mg/L; vancomycin, 10 mg/L; polymyxin B, 2.5 IU/L, amphotericin B, 2.5 mg/L (Sigma-Aldrich, St. Louis, MO). All *H*. *pylori* cultures were grown at 37°C under microaerophilic conditions of 5% O_2_ generated by an Anoxomat (Advanced Instruments, Norwood, MA).

**Table 1:** *Helicobacter pylori* strains used to infect airway epithelial cell cultures.

Strain	Description	Antibiotic Resistance^1^	Source
J166	Wild type clinical isolate		[26]
PMSS1	Wild type clinical isolate		[27]
PMSS1Δ*cagA*	PMSS1 with entire *cagA* plus 590 bp upstream and 168 bp downstream replaced by CAT_rpsL casette	Cm	[28]
PMSS1*ΔcagY*	PMSS1 with bp 13–5814 of *cagY* replaced by CAT_rpsL casette	Cm	[28, 29]

^1^Cm, chloramphenicol

### *In vitro* epithelial cell *H*. *pylori* infection

After 20–24 hr of agar culture incubation, *H*. *pylori* was suspended in brucella broth at MOI of 1, 10 or 100 and subsequently added to 16-HBE or ALI primary airway epithelial cell cultures at MOI of 1 or 10 only. Brucella broth served as the negative control or MOI of 0. Cultures were washed in basal airway epithelial cell media that did not contain antibiotics or growth supplements (BEBM, Lonza, Walkersville, MD) prior to addition of *H*. *pylori*. For ALI primary cultures, bacteria were added directly to the apical side of the transwell. At 24 hr post-infection, supernatants from apical surfaces were collected for cytokine analysis. Where indicated, primary cultures were also treated with heat-killed *H*. *pylori* (10^8^ cells/ml, Invivogen, San Diego, CA).

### Quantification of proinflammatory cytokines

IL-8 (R&D Systems, Minneapolis, MN) and IL-6 (eBiosciences, San Diego, CA) protein was measured by ELISA, per the manufacturer’s instructions. The limit of detection for ELISA assays was 2 pg/mL (IL-6), and 15.625 pg/mL (IL-8).

### Western blot

HBE cells were lysed and processed for western blot analysis as previously described for the human gastric adenocarcinoma (AGS) cell line [25]. Phosphorylated CagA was detected by chemiluminescent immunodetection with a mouse anti-phosphotyrosine IgG (PY99) (Santa Cruz Biotechnology, Santa Cruz, CA), followed by stripping and chemiluminescent immunodetection with a rabbit anti-CagA IgG (Austral Biological, San Ramon, CA).

### Quantitative RT-PCR

In brief, total RNA was isolated from primary airway epithelial cell cultures using TRIzol reagent (Invitrogen, ThermoFisher, Scientific). Samples were treated with DNase I to remove DNA contamination as described by the manufacturer (Applied Biosystems, Carlsbad, CA). cDNA was synthesized by using 500 ng of RNA to make 50μL of cDNA using random hexamer primers and High Capacity MultiScribe™ Reverse Transcriptase kit (Applied Biosystems, Carlsbad, CA) as previously described [20, 23]. NOD1 (rh02859615_m1) and B2M (rh02847368_m1) mRNA were measured by qRT-PCR with commercially available rhesus-specific primer-probe assays (Invitrogen, ThermoFisher Scientific) and detected using the Applied Biosystems Viia™7 Real-Time PCR system. Cycle threshold (Ct) for NOD1 were normalized to B2M for each sample in duplicate to determine 2^-ΔCt^.

### NOD1 agonist and inhibitor treatment

Airway epithelial cells were cultured under ALI conditions as described and washed with BEBM before treatment. Cells were treated with iE-DAP (50ug/ml, Invivogen, San Diego CA), iE-LYS (50ug/ml; Invivogen, San Diego CA) or negative control media for 24 hr prior to supernatant harvest. For NOD1 inhibition, cells were pretreated with ML130 (1uM, Selleckchem, Houston, TX) for 1hr at 37°C, 5% CO_2_ before addition of iE-DAP or *H*. *pylori*. Similar procedures were performed using NF-kappaB inhibitor JSH-23 (10uM, ApexBio Tech, TX) and p38 MAP kinase inhibitor SB203580 (10uM, Selleckchem, Houston, TX) prior to addition of *H*. *pylori*.

### Statistics

Data are reported as mean ± standard error of the mean (SEM), unless otherwise noted. Bacteria concentration-dependent responses and age-dependent differences were evaluated for each strain separately by one-way analysis of variance (ANOVA) followed by Bonferroni multiple comparison test (post-test). Unpaired t-test and Repeated Measure (RM) one-way ANOVA were also used where appropriate. GraphPad Prism 7 (GraphPad, La Jolla, CA) was used for statistical analysis. Specific *p* values are listed in the figures.

## Results

### Induction of proinflammatory cytokine synthesis in a human bronchial epithelial cell line by *H*. *pylori* is dependent upon strain and MOI

**Fig 1:**
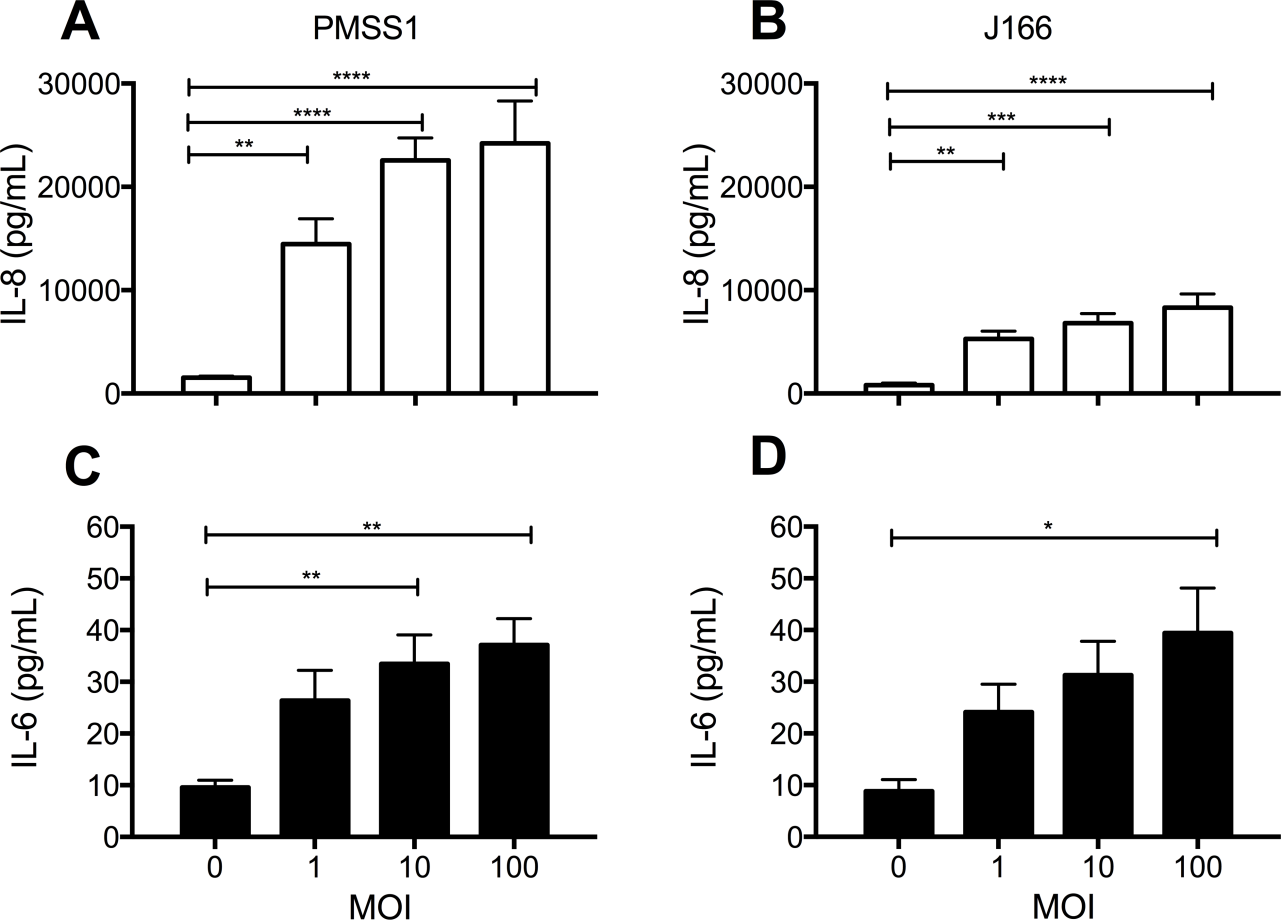
*H*. *pylori* infection can induce IL-8 and IL-6 synthesis in the 16-HBE human bronchial epithelial cell line. Supernatants from 16-HBE cell cultures were collected following infection with either *H*. *pylori* strain PMSS1 or J166. **(A and C)** Effect of 24 hr incubation with PMSS1 on IL-8 (n = 9) and IL-6 (n = 9) protein concentration. **(B and D)** Effect of 24 hr incubation with J166 on IL-8 (n = 10) and IL-6 (= 7) protein concentration. Statistical significance was determined by one-way ANOVA followed by Bonferroni’s multiple comparison test. Error bars represent SEM. *p<0.05, **p<0.01, ***p<0.001 ****p<0.0001

To initially assess whether *H*. *pylori* can directly induce innate immune responses in airway epithelium, we infected the human bronchial epithelial cell line 16-HBE with either strain PMSS1 or strain J166 for 24 hours, followed by assessment of secreted proinflammatory cytokine synthesis. We found that induction of IL-8 and IL-6 by 16-HBE cell cultures following addition of bacteria was dependent upon *H*. *pylori* strain and MOI (Fig 1). As shown in Fig 1A and 1B, PMSS1 and J166 induced IL-8 synthesis in a MOI-dependent manner (p<0.0001 by one-way ANOVA for both PMSS1 and J166), with a higher concentration of cytokine production attributed to the PMSS1 strain. IL-6 synthesis was also significantly dependent upon MOI (PMSS1 p<0.01 by one-way ANOVA; J166 p<0.05 by one-way ANOVA), but both *H*. *pylori* strains induced comparable levels of cytokine synthesis in 16-HBE cell cultures following infection (Fig 1C and 1D).

### Induction of IL-8 protein synthesis by *H*. *pylori* is dependent upon chronological age of primary airway epithelial cell cultures

**Fig 2:**
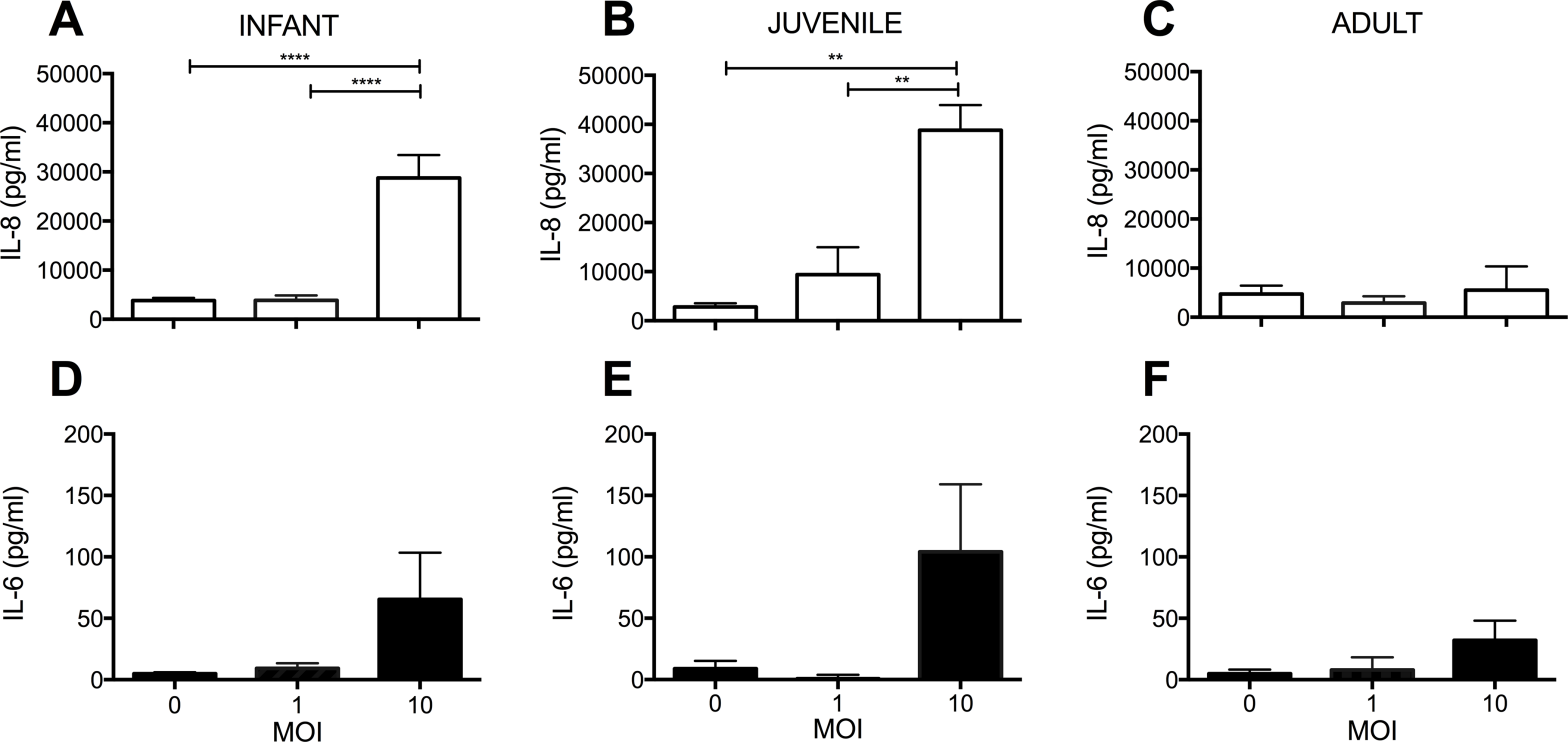
Age-dependent IL-8 synthesis in primary airway epithelial cell cultures following *H*. *pylori* infection. Supernatants from infant, juvenile or adult monkey primary airway epithelial cell cultures were collected following infection with *H*. *pylori* strain PMSS1. **(A and D)** Effect of 24 hr incubation with *H*. *pylori* PMSS1 on IL-8 and IL6 protein concentration in infant cultures (n = 10). **(B and E)** Effect of 24 hr incubation with *H*. *pylori* PMSS1 on IL-8 and IL-6 protein concentration in juvenile cultures (n = 3). **(C and F)** Effect of 24 hr incubation with *H*. *pylori* PMSS1 on IL-8 and IL-6 protein concentration in adult cultures (n = 4). Statistical significance was determined using one-way ANOVA followed by Bonferroni’s multiple comparison test. Error bars represent SEM. ** p <0.01, **** p< 0.0001

Following verification that *H*. *pylori* can promote proinflammatory cytokine synthesis in a human bronchial epithelial cell line, we next determined whether primary cultures of tracheobronchial airway epithelial cells can respond in a similar fashion. We focused our analysis with primary cultures using the PMSS1 strain based upon our initial finding of a robust cytokine response in the 16-HBE cell line relative to the J166 strain (Fig 1). Primary cultures derived from infant, juvenile and adult monkeys were infected with PMSS1 for 24 hours, followed by measurement of IL-8 and IL-6 protein synthesis. We found that IL-8 synthesis by primary cultures in response to PMSS1 infection was dependent upon age and MOI (Fig 2). Both infant (p<0.0001 by one-way ANOVA) and juvenile (p<0.01 by one-way ANOVA) primary cultures (Fig 2A and 2B) synthesized IL-8 in a MOI-dependent manner with *H*. *pylori* infection, whereas we did not observe significant differences in adult primary cultures under identical conditions (Fig 2C). There was a modest increase in IL-6 synthesis at MOI = 10 relative to controls for infant and juvenile primary cultures, but this did not reach statistical significance by one-way ANOVA (Fig 2D–2F).

### The *H*. *pylori* type IV secretion system (T4SS) is required for optimal IL-8 synthesis in airway epithelium

**Fig 3:**
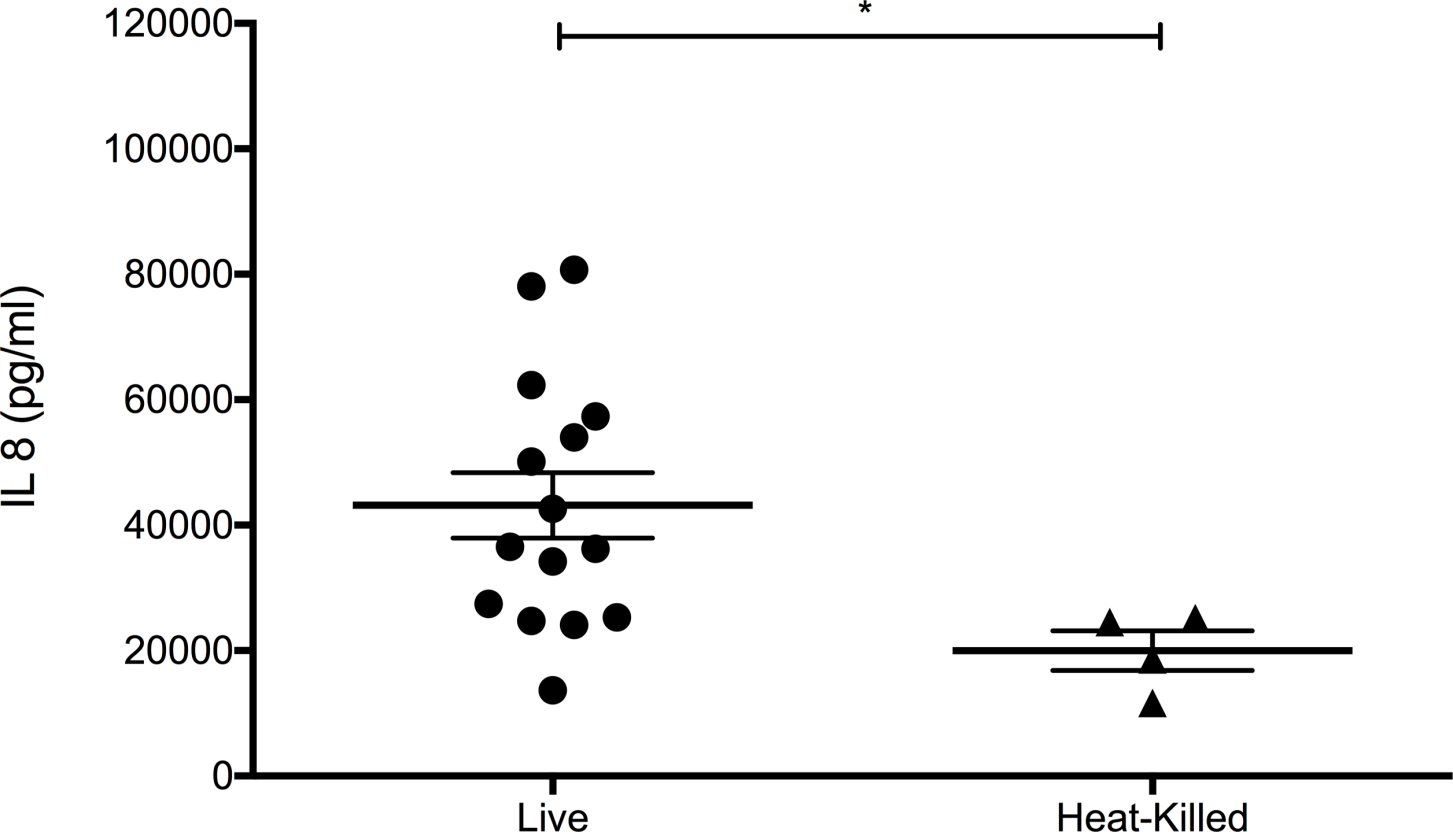
*H*. *pylori* viability is essential for optimal IL-8 synthesis in primary airway epithelial cell cultures. Supernatants from juvenile primary airway epithelial cell cultures were collected following infection with **(A)** *H*. *pylori* PMSS1 (MOI = 2.5–15) for 24 hr or treatment with **(B)** 10^8^ cells/ml heat-killed *H*. *pylori* for 24 hr. Statistical significance was determined using unpaired t-test *p<0.05. Error bars represent SEM.

To investigate the cellular and molecular mechanisms by which *H*. *pylori* can directly elicit proinflammatory cytokine synthesis in airway epithelium, we first assessed whether viable bacteria are required for IL-8 synthesis in primary cultures of juvenile tracheobronchial airway epithelial cells. When compared with heat-killed *H*. *pylori*, induction of IL-8 was significantly more robust following infection of primary airway epithelial cell cultures with live organisms, suggesting the requirement of an active process (Fig 3).

**Fig 4:**
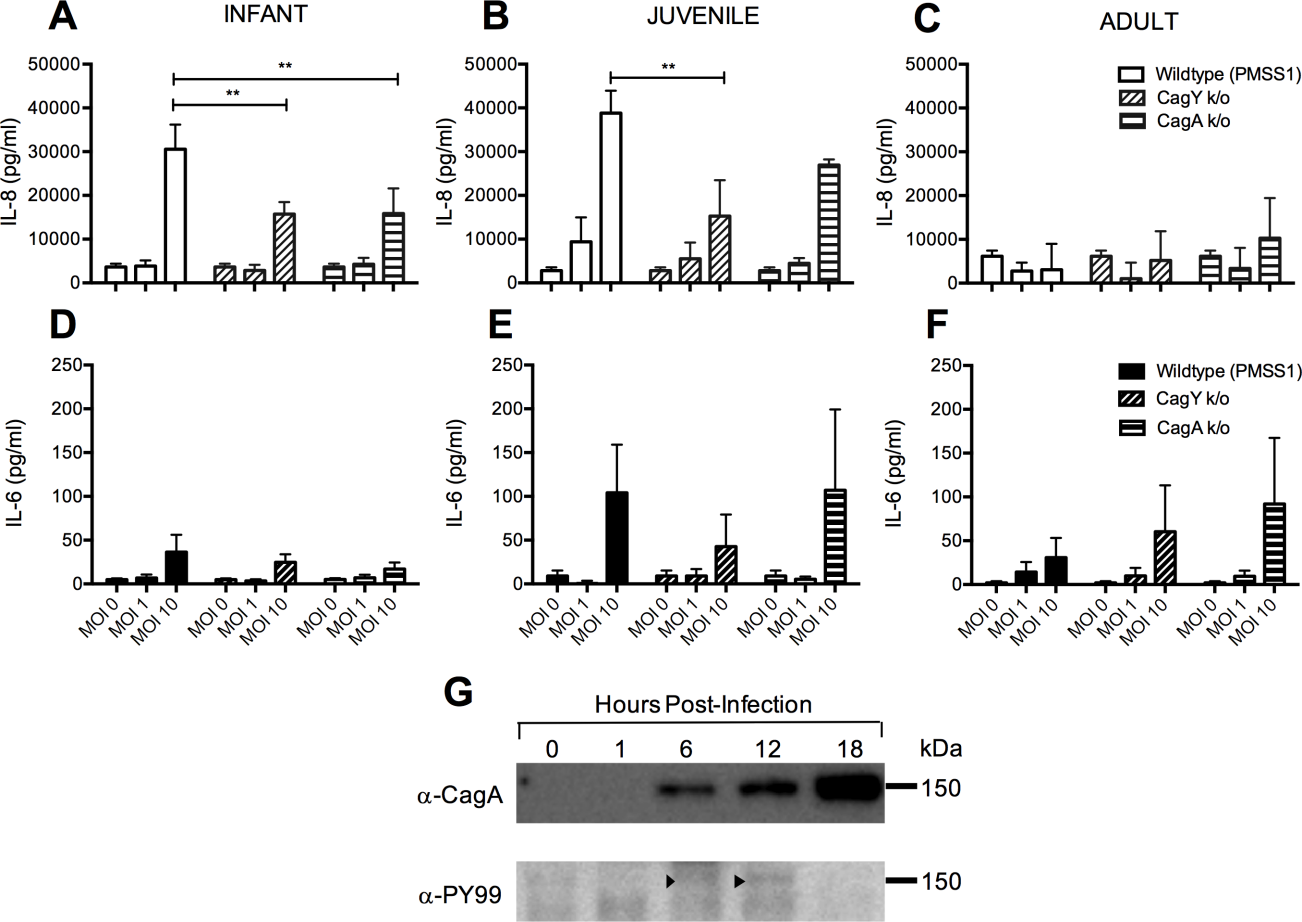
Effect of *cag*PAI mutation on H. pylori-induced proinflammatory cytokines in primary airway epithelial cell cultures. Primary airway epithelial cell cultures were infected with PMSS1 WT, *cagY* knockout or *cagA* knockout strains for 24 hr, followed by collection of supernatant for ELISA. **(A and D)** Effect of WT or mutant stains on IL-8 and IL-6 protein concentration in cultures of infant epithelial cells (n = 3). **(B and E)** Effect of WT or mutant stains on IL-8 and IL-6 protein concentration in cultures of juvenile airway epithelial cells (n = 3). **(C and F)** Effect of WT or mutant strains on IL-8 and IL-6 protein concentration in cultures of adult airway epithelial cells (n = 3). Statistical significance was determined by two-way ANOVA followed by Bonferroni’s multiple comparison test. Error bars represent SEM. **p < 0.01 (G) Western blot analysis for PMSS1 CagA phosphorylation following infection of the 16-HBE cell line. 16-HBE cell cultures were assessed at 0, 1, 6, 12 and 18 hrs post-infection with PMSS1 (MOI = 10). Arrows point to detected anti-phosphotyrosine labelled bands at 150 kDa for 6 and 12 hrs.

Clinical disease is observed more commonly in individuals infected with *H*. *pylori* strains carrying the *cag*PAI [25]. Thus, we proceeded to define the role of the *cag*PAI in the cytokine response elicited by *H*. *pylori* infection of primary tracheobronchial airway epithelial cell cultures using mutants of the *H*. *pylori* PMSS1 strain that contain deletions of either *cag*A, which encodes the CagA oncoprotein, or *cag*Y, which encodes an essential gene for the *H*. *pylori* T4SS. At the highest concentration of bacteria tested (MOI = 10), we found that synthesis of IL-8 in infant primary cultures were significantly reduced following infection with *cag*PAI mutant strains (both *cag*Y and *cag*A) when compared to the wildtype (WT) strain (p<0.01 WT vs *cag*Y, WT vs *cag*A) (Fig 4A). In juvenile primary cultures, significant differences were observed between the WT and *cag*Y mutant strains at MOI = 10 (p<0.01) (Fig 4B), while infection with WT or any of the two mutant strains showed no difference in adult primary cultures (Fig 4C). Compared with IL-8, global synthesis of IL-6 was much less for all strains evaluated but there was a significant effect of MOI for infant (p<0.01) and juvenile primary cultures (p<0.05) (two-way ANOVA, MOI vs strain) (Fig 4D–4F). Additional evidence of CagA delivery to the airway epithelium was provided by western blot analysis, which detected the presence of phosphorylated CagA at 6 and 12 hours post-infection of the 16-HBE cell line with PMSSI (WT) (Fig 4G). CagA was also detected at 18 hours post-infection, however there was no detectable signal for phosphotyrosine.

### NOD1 activation induces IL-8 synthesis in primary airway epithelial cell cultures

**Fig 5:**
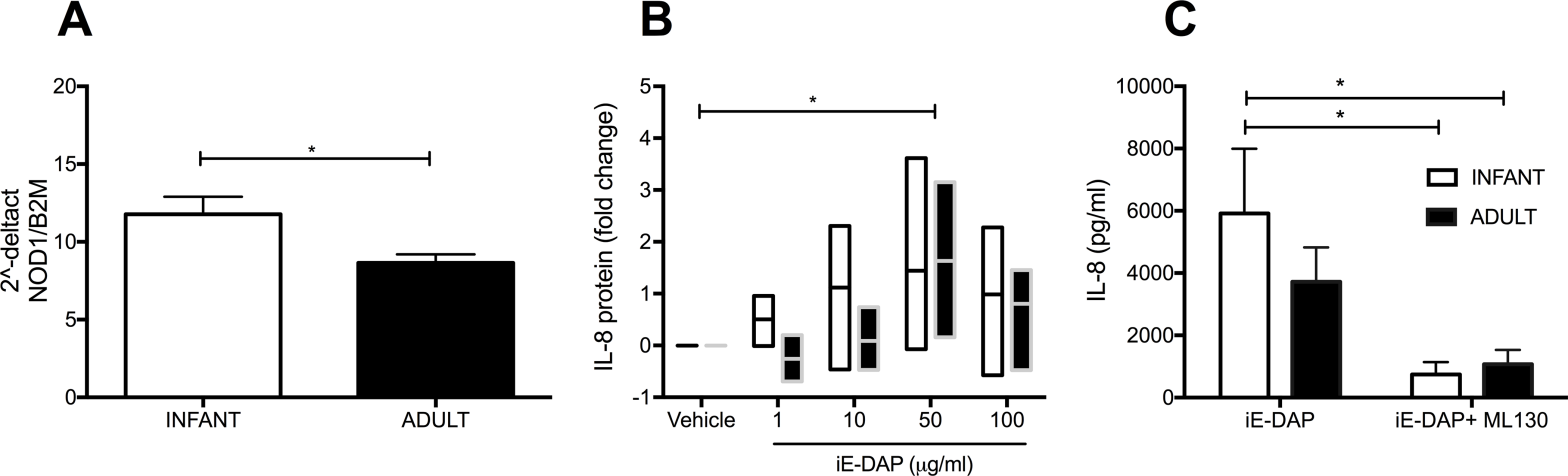
Activation of NOD1 promotes IL-8 synthesis in both infant and adult airway epithelial cell cultures. **(A)** NOD1 real time PCR analysis for both infant (n = 4) and adult (n = 9) primary cultures. *p<0.05 as determined by unpaired t-test. **(B)** Fold change of IL-8 protein in infant (n = 5) or adult (n = 3) primary cultures following treatment with iE-DAP. *p<0.05 by two-way ANOVA followed by Bonferroni’s multiple comparison test. **(C**) IL-8 protein synthesis in infant (n = 8) or adult (n = 9) primary cultures following treatment with iE-DAP only or iE-DAP in the presence of ML130. *p<0.05 by two-way ANOVA followed by Bonferroni’s multiple comparison test, infant iE-DAP vs infant ML130, infant iE-DAP vs adult ML130. Error bars represent SEM.

Because *H*. *pylori* uses T4SS to transport peptidoglycan inside host cells [30], in addition to CagA [31] and DNA [32], we determined whether the cytosolic peptidoglycan sensor NOD1 plays a role in promoting IL-8 synthesis in airway epithelium. Based upon our initial finding that airway epithelium from young animals was more sensitive to the proinflammatory effects of *H*. *pylori*, we first assessed for differential NOD1 expression; infant primary cultures expressed more NOD1 mRNA in comparison with adult primary cultures (p<0.05) (Fig 5A). Using the NOD1 agonist, gamma-D-glutamyl-meso-diaminopimelic acid or iE-DAP, a soluble component of bacterial peptidoglycan, we found that both infant and adult primary cultures responded to iE-DAP in a dose-dependent manner (p<0.01 by two-way ANOVA), with a significant increase in IL-8 protein peaking at 50 ug/ml (Fig 5B). Treatment of infant and adult primary cultures with a selective NOD1-inhibitor ML130 prior to iE-DAP activation resulted in almost a complete abrogation of IL8 synthesis (p<0.01 by two-way ANOVA) (Fig 5C).

### *H*. *pylori*-mediated synthesis of IL-8 is NOD1-NF-κB independent and p38-MAPK dependent

**Fig 6:**
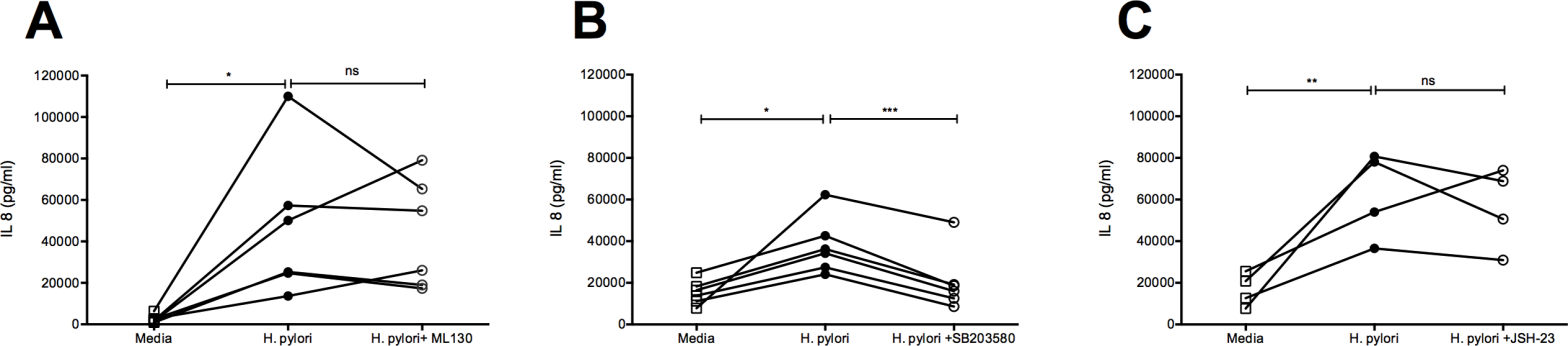
*H*. *pylor*i induced IL-8 synthesis is inhibited by the p38 MAP kinase inhibitor SB203580. Juvenile primary cultures were infected with *H*. *pylori* (PMSS1) in the presence of inhibitors for NOD1, NF-kappaB and p38 kinase. Supernatant were harvested after 24hr. **(A)** *H*. *pylori*-induced IL-8 synthesis following treatment with ML130 (n = 6). *p<0.05 (*H*. *pylori* vs media). **(B)** *H*. *pylori*-induced IL-8 synthesis following treatment with JSH-23 (n = 4). **p<0.01 (*H*. *pylori* vs media). **(C)** *H*. *pylori*-induced IL-8 synthesis following treatment with SB203580 (n = 6). *p<0.05 (*H*. *pylori* vs media), ***p<0.001 (*H*. *pylori* vs. *H*. *pylori* + SB203584). Statistical significance was determined by repeated measures one-way ANOVA.

*H*. *pylori* is known to activate NOD1 signaling events through the NF-kappaB pathway in gastric epithelium [33]. We next examined whether induction of IL-8 synthesis by *H*. *pylori* is mediated through activation of the NOD1-NF-kappaB signaling pathway using the inhibitors ML130 to block NOD1 and JSH-23 to block NF-kappaB signaling. *H*. *pylori* infection of juvenile primary cultures induced a significant IL-8 response which was only slightly reduced in 3 out of 5 animals following treatment of cultures with ML130; there was no statistical difference between ML130 treatment and vehicle controls (Fig 6A). Identical experiments using JSH-23 treatment similarly resulted in 3 out of 4 animals displaying a modest reduction in IL-8 synthesis without reaching statistical significance (Fig 6B). As an alternative to the NF-kappaB pathway, we subsequently determined whether the p38-MAPK pathway may play a role in the induction of IL-8 synthesis following *H*. *pylori* infection in airway epithelium. Inhibition of p38 MAP kinase by treatment of juvenile primary cultures with SB203580 resulted in a significant reduction of IL-8 synthesis following infection with *H*. *pylori* (Fig 6C).

## Discussion

Using an *in vitro* infection model, our study shows that the gastric pathogen *H*. *pylori* can directly elicit an innate immune response by lung epithelium, using a human airway epithelial cell line and rhesus monkey primary airway epithelial cells. For the airway cell line assessed, induction of IL-8 and IL-6 protein synthesis by *H*. *pylori* was significant at MOI = 1. Primary airway epithelial cells were less sensitive to *H*. *pylori* infection, requiring an MOI = 10 to elicit a comparable induction of IL-8 synthesis with a minimal effect on IL-6 synthesis. Our initial finding of *H*. *pylori* strain variation in innate immune induction suggested that the *cag*PAI may play a role in production of cytokine by airway epithelium, which was supported by experiments utilizing *cag*Y and *cag*A mutant *H*. pylori strains. A striking result was the observation that chronological age of airway epithelium had a significant effect on susceptibility to *H*. *pylori* induced inflammation; only airway epithelial cells derived from infant or juvenile animals produced a IL-8 robust response to *H*. *pylori* infection. While both NOD1/NF-kappaB and p38 MAP kinase pathways have been reported for *H*. *pylori* mediated inflammation in gastric epithelium, IL-8 induction by *H*. *pylori* infection of airway epithelium was predominantly mediated by the p38 MAP kinase pathway.

*H*. *pylori* is a gastrointestinal tract pathogen and is adapted to live in the gastric epithelium. *H*. *pylori* uses several virulence mechanisms involving the *cag*PAI to establish chronic infection in the gastric mucosa. Two factors that have been shown to be important in the contribution of the *cag*PAI in infection are *cag*A and components of T4SS, which inject the CagA protein, peptidoglycan, and DNA into host epithelial cells [30–32]. Our results indicate that *H*. *pylori*-induced expression of proinflammatory cytokines is strain-dependent, with a greater response in strain PMSS1 which we [28] and others [34] recently showed generate multiple copies of *cag*A. Moreover, deletion of *cag*A had a deleterious effect on IL-8 synthesis in infant and juvenile airway epithelial cells (Fig 4). The IL-8 response to the *cag*Y deletion mutant for PMSS1 was also significantly reduced in infant airway epithelial cells. We have recently reported that *cag*Y is a critical component of the T4SS system that injects CagA, therefore *cag*A may be most critical gene for promoting the IL-8 response in airway epithelium [29].

As additional support for the requirement of the Type IV secretion system for *H*. *pylori* responsiveness, IL-8 expression by airway epithelial cells appeared to be dependent upon intact and viable bacteria (Fig 3). Western blot analysis indicated phosphorylation of a ~150 kDa protein corresponding to the molecular weight of CagA at 6 and 12 hours post-infection in the 16-HBE cell line, providing evidence of CagA delivery into airway epithelium. Phosphorylation of CagA was not detected at 18 hours post-infection in airway epithelium, despite detectable CagA protein by immunoblot; it is possible that other products of *H*. *pylori* may contribute toward the production of IL-8 in our system. For example, *H*. *pylori* secreted peptidyl prolyl cis, trans-isomerase can elicit cytokine responses in immune cells, suggesting that potential immunomodulatory mechanisms for *H*. *pylori* are diverse with regard to cellular targets and pathways [35]. A recent study has shown that VacA, an *H*. *pylori* exotoxin, is detectable in human lung biopsy specimens and can directly stimulate the secretion of inflammatory cytokines from lung airway epithelial cells *in vitro* [6]. VacA has also been reported to be a critical determinant for *H*. *pylori*-mediated protection from multiple parameters of the allergic asthma phenotype in a rodent model, lending further support for immunomodulatory properties of *H*. *pylori* in the context of the lung [36]

Interaction of the microbe with gastric epithelium results in activation of various cell signaling pathways including NF-kappaB [33, 37]. In our study, we directly added *H*. *pylori* on the apical surface of the airway epithelial cells and measured IL-8 to assess for NF- kappaB -dependent and independent pathways. Furthermore, we tested the signaling pathways by using specific inhibitors to block NOD1/NF-kappaB and p38 pathways. In experiments using ML130 and JSH-23, inhibitors to NOD1 and NF-kappaB, respectively, there was a modest reduction in IL-8 production in airway epithelial cell cultures but this did not reach statistical significance. It is feasible that alternative pharmacological strategies may elicit a more robust response in our culture system, particularly for NF-kappaB. However, IL-8 synthesis was significantly blocked by inhibition of the p38 MAP kinase pathway using the inhibitor SB203580. Indeed, studies using AGS gastric epithelial cells indicate that upon co-culture with *H*. *pylori*, activation of members of MAP kinases are evident within 5 minutes, resulting in an increase of IL-8 production [37]. In airway epithelial cells, IL-8 synthesis can be mediated by both NF-kappaB dependent and independent pathways, with evidence of post-transcriptional regulation by p38 [38].

The route of gastrointestinal infection by *H*. *pylori* is controversial, however the microbe has been cultured from vomitus and saliva [39, 40]. *H*. *pylori* can be cultured from the air near a vomiting subject and has been detected in the lung of COPD affected patients using PhyloChip analysis, suggesting that it can be aspirated into the airways [41, 42]. These data suggested that while *H*. *pylori* resides in the stomach, it is indeed capable of reaching the oral cavity and becoming aspirated, enabling access to the upper and lower airways and potentially inducing an immune response from the airway epithelium. Given the inhospitable growth environment of the lung for *H*. *pylori*, it is most likely that the interaction of viable bacteria with airway epithelia is brief. Although we did not conduct a time course for our culture studies, our data show that induction of IL-8 synthesis takes place within 24 hours of infection.

Our previous studies have reported that the infant airway epithelium displays a hyporesponsive innate immune phenotype after endotoxin exposure and attenuated expression of IL22R1, a relevant marker for both immune defense and lung repair form injury compared to adults [20, 23]. In contrast, the current findings indicate a heightened innate immune response in the infant airway epithelial cultures compared to adults after *H*. *pylori* infection, suggesting that the TLR4 pathway was not required. Although *H*. *pylori* is a gram-negative bacterium (with an outer membrane containing lipopolysaccharide), our findings with strain as well as *cag*A/*cag*Y dependence suggests that the Type IV Secretion system may be more important in eliciting an epithelial cell response in the lung for microbes that utilize this strategy.

Further studies are needed to elucidate the downstream events that take place upon the interaction of *H*. *pylori* with the airway epithelial cells, as well as evaluate additional immunomodulatory products of *H*. *pylori*. In particular, *in vivo* assessment of the efficacy for *H*. *pylori* as a protective mechanism in childhood asthma is a logical next step. Understanding how *H*. *pylori* induces innate immune response in the airway epithelial cells may help elucidate the protective mechanisms of microbiota in the respiratory mucosa, ultimately leading to prevention of chronic disease.

## Supporting information

S1 Supplementary Spreadsheet Raw data for Figs 1–6. (XLSX)
